# Pituitary Metastasis of Pulmonary Large Cell Neuroendocrine Carcinoma: A Case Report

**DOI:** 10.7759/cureus.7226

**Published:** 2020-03-09

**Authors:** Hsin-pei Hu, Arpita Sengupta, David Bowes

**Affiliations:** 1 Radiation Oncology, London Regional Cancer Program, Western University, London, CAN; 2 Radiation Oncology, Nova Scotia Cancer Centre, Queen Elizabeth II Health Sciences Centre, Halifax, CAN; 3 Radiation Oncology, Dalhousie University, Halifax, CAN; 4 Radiation Oncology, Queen Elizabeth II Health Sciences Centre, Halifax, CAN

**Keywords:** large cell neuroendocrine carcinoma, lung cancer, pituitary metastasis, intracranial metastasis, pituitary tumor, transsphenoidal surgery, stereotactic radiotherapy, diabetes insipidus

## Abstract

The pituitary gland is an uncommon site of tumor metastasis in the brain, comprising only 1% of all intracranial metastasis. Large cell neuroendocrine carcinoma (LCNEC) is similarly rare, accounting for only 3% of all lung malignancies in adults. We describe a case of LCNEC of lung origin that metastasized to the pituitary gland. The pituitary lesion was found during the workup for a metastatic LCNEC of lung origin in the ovary. Initially thought to be a pituitary adenoma, interval growth of the lesion during imaging follow-up raised clinical suspicion of a second metastatic site. The patient underwent endoscopic resection and pathological examination confirmed the pituitary lesion to be from the lung primary. Post-operatively, the patient developed signs and symptoms of diabetes insipidus that was adequately treated with DDAVP. The patient underwent postoperative radiotherapy one month after the surgery and a repeat MRI at the 12-month follow-up demonstrates no progression of the pituitary lesion.

## Introduction

Tumor metastasis to the pituitary gland is uncommon, and found in only 1% of all intracranial metastasis and 2% of all autopsied cancer cases [1]. Pulmonary large cell neuroendocrine carcinomas (LCNEC) are rare, account for approximately 3% of all lung malignancies in adults [2]. Due to the relative rarity of pituitary metastases and pulmonary LCNEC, there have been no reported cases describing a presentation of LCNEC metastasis in the pituitary gland. We present the diagnosis and treatment of a patient with LCNEC of lung origin that metastasized to the pituitary gland.

## Case presentation

A 60-year-old woman with a 40-pack-year (former) smoking history was diagnosed with pT1b N1 M0 high grade LCNEC in 2013. Comorbidities include hypothyroidism, migraines, depression, anxiety, and fibromyalgia. She was treated with lobectomy and concurrent adjuvant chemoradiation with 40 Gy in 15 fractions of radiotherapy to the chest with four cycles of cisplatin and etoposide.

**Figure 1:**
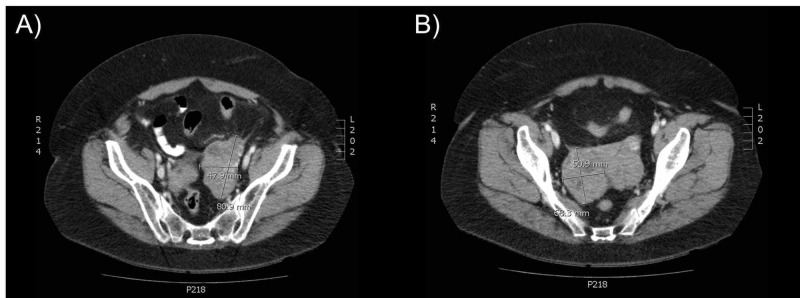
CT images of ovarian masses found five years after initial diagnosis and treatment of lung LCNEC. The patient presented with bilateral ovarian masses and neurological symptoms during follow-up surveillance. Images reveal bilateral ovarian masses: (A) Left: 8.1 x 4.8 cm; (B) Right: 6.8 x 5.1 cm. Surgical resection confirmed these masses as LCNEC of pulmonary origin. LCNEC: Large cell neuroendocrine carcinoma

**Figure 2:**
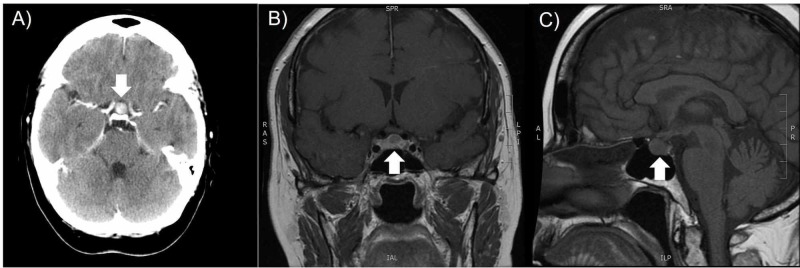
CT and MRI T1-FLAIR images of the pituitary tumor found five years after initial diagnosis and treatment of lung LCNEC. The patient presented with neurological symptoms including unsteady balance, bilateral temporal headaches, memory loss, and mild mood alterations. Images reveal an enhancing pituitary tumor, initially thought to be a pituitary adenoma, measuring 5 mm size with suprasellar extension near the optic chiasm (arrow). (A) CT axial view; (B) MRI T1-FLAIR coronal view; (C) MRI T1-FLAIR sagittal view. LCNEC: Large cell neuroendocrine carcinomas

In January 2018, the patient presented with bilateral ovarian masses and developed severe fatigue, dizziness, unsteady balance, bilateral temporal headaches, memory loss and mild mood alterations. Staging workup revealed bilateral ovarian masses of size 8.1 x 4.8 cm and 6.8 x 5.1 cm on CT scan and a CA-125 level of 114 (Figure 1). Subsequent total abdominal hysterectomy, bilateral salpingo-oophorectomy, omentectomy and staging of the ovarian masses tested positive for LCNEC of pulmonary origin. The staging CT scan and MRI images also revealed a 5-mm lesion in the pituitary gland that was initially thought to be a pituitary adenoma (Figure 2). The follow-up head CT scan two months later revealed interval growth of the pituitary lesion to 9 mm. The physical examination revealed no departure from her baseline neurological functioning and vision, which includes fatigue, daily headaches, slight blurry vision and longstanding floaters in her vision. Including her hypothyroidism that was maintained with levothyroxine 50 mcg, her hormone levels were within the normal range. She was scheduled for an endoscopic image-guided transsphenoidal excision of the pituitary lesion on suspicion of a metastasis.

**Figure 3:**
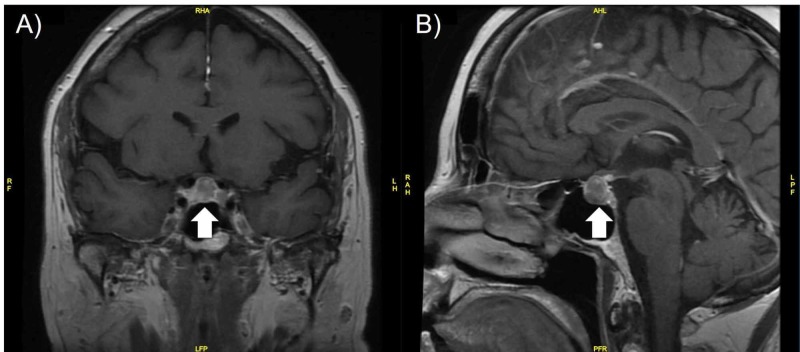
MRI T1-FLAIR images of the pituitary tumor prior to transsphenoidal resection. Images reveal an inhomogeneously enhancing pituitary tumor measuring 10 mm size with suprasellar extension and contacting the inferior aspect of the optic chiasm (arrow). (A) Coronal view; (B) Sagittal view.

The preoperative magnetic resonance imaging showed a 10-mm mass in the sellar and suprasellar region (Figure 3). Her preoperative hormonal evaluation, complete blood count, electrolytes, renal and liver function tests were within normal range. The patient underwent uncomplicated endoscopic image-guided transsphenoidal subtotal excision of the pituitary lesion. Postoperatively, the patient developed polyuria, hypernatremia, and low urine specific gravity that responded to desmopressin administration. These signs and symptoms of diabetes insipidus were treated and maintained with desmopressin 60 mcg.

**Figure 4:**
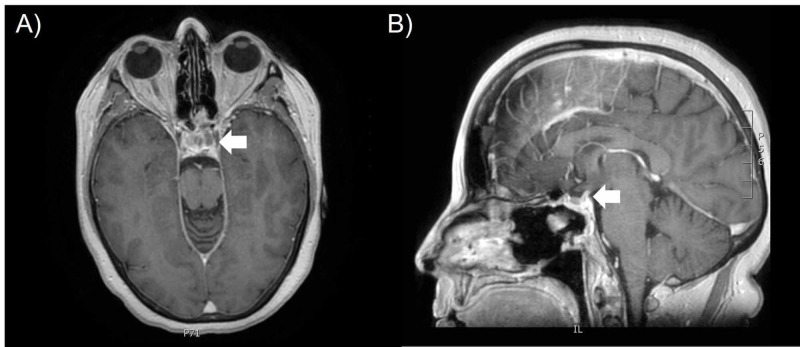
MRI T1-FLAIR images of the pituitary tumor after transsphenoidal resection and prior to radiation treatment. The patient underwent transsphenoidal resection of the anterior wall, sphenoid sinus, posterior nasal septum, posterior ethmoid sinuses prior to stereotactic radiotherapy. There is some low signal within the sella on the left side that may represent residual tumor (arrow). No mass is seen in the suprasellar region or cavernous sinus. (A) Axial view; (B) Sagittal view.

The patient was referred for postoperative radiotherapy. At the time of consultation, she reported fatigue with poor appetite, daily headaches, and severe nausea with movement. On post-resection MRI, there was an area of small hypodensity within the sella on the left side which was suspicious for residual tumor (Figure 4). There was no extension to the cavernous sinus. The patient was discussed at the stereotactic radiotherapy tumor board rounds and the recommendation was made to offer radiotherapy. The patient underwent stereotactic radiotherapy (25 Gy in five fractions with 6 MV photon beams). The clinical target volume (CTV) included the area of suspected residual tumor as seen on diagnostic and treatment planning MRI, as well as the resection cavity in the sella turcica. A 2-mm margin was added to the CTV to generate a planning target volume (PTV). The dose was prescribed to the 90% isodose level and the treatment plan was designed to achieve coverage of the PTV with the 90% isodose line. She was on 4 mg dexamethasone and anti-emetics during treatment. Her nausea had improved by treatment completion. The patient has had repeat MRIs for follow-up which demonstrate no progression of the pituitary lesion at 12 months after the initial surgery.

## Discussion

The presented case is a rare presentation of LCNEC of lung origin that metastasized to the pituitary gland. A systematic review has found the incidence of pituitary metastases to be approximately 0.9% among all intracranial metastases and 1.9% among all autopsied cancer cases [1]. The most common origins of primary tumors metastatic to the pituitary are breast and lung, but there have been reports of other primary sites including prostate, kidney, gastrointestinal tract and thyroid [3]. Based on careful literature review, there has been no reported case of pituitary metastasis with pulmonary LCNEC origin.

LCNECs are rare pulmonary tumors that are classified as a variant of non-small cell lung cancer, but share similar clinical behavior, morphology, and prognosis as small cell lung cancer (SCLC) [2]. LCNECs have similar gene expression profiles and some common chromosomal alterations as SCLCs, but are separate entities from SCLCs due to differences in expression of CK7, CK18, E-cadherin, and beta catenin [4,5]. Nonetheless, LCNECs have a high distant metastasis rate of 65% similar to SCLC, including a reported 32% brain metastasis rate [6,7]. Although there is no established therapeutic approach, primary surgery should be considered for all surgical candidates, and based on the poor outcomes with surgery alone, systemic therapy could be considered in the adjuvant setting as was done in this case [2]. Due to the nodal disease and lack of surgical staging of the mediastinum, the decision was made to offer adjuvant radiotherapy as well. In the setting of brain metastases, surgery and radiotherapy are considered acceptable palliative approaches [8]. For advanced LCNEC, platinum-/etoposide-based chemotherapy regimens that are applied for SCLCs appear to offer advantages in survival [7,9].

The main routes of metastasis to the pituitary are hematogenous spread and direct invasion from the skull base [3]. It is theorized that the direct arterial blood supply of the posterior pituitary is a factor for the higher prevalence of posterior pituitary involvement compared to the anterior pituitary, which receives its blood supply from the hypophysial portal system [10]. Preferential dissemination to the posterior pituitary may be related to the most common presenting symptom of pituitary metastases - diabetes insipidus. While clinical and pathological review estimates only 7% of pituitary metastases are symptomatic, some studies report as high as 70% of pituitary metastases include diabetes insipidus as the presenting symptom [11,12]. Other symptoms such as cranial nerve palsies, anterior hypopituitarism, visual disturbances, and headaches may also be present [1,11,12]. Due to the high incidence in pituitary metastases, rapid onset of diabetes insipidus and/or cranial nerve palsies should raise clinical suspicion of pituitary metastases rather than pituitary adenoma [13].

Some authors have suggested some non-specific imaging features of pituitary lesions may help distinguish a metastatic lesion from a pituitary primary. Pituitary metastases typically exhibit fast growth. Other notable MRI features of pituitary metastases include relative isointensity to brain on both T1- and T2-weighted images, thickening of the pituitary stalk, invasion of the cavernous sinus, and sclerotic changes around the sella turcica [13-15]. The presence of other intracranial lesions should also raise clinical suspicion of a non-pituitary origin in a pituitary lesion.

Symptomatic pituitary metastases are most often treated locally by surgery and radiotherapy. Transsphenoidal surgery is the preferred surgical approach for debulking and symptom relief [16]. For nonsurgical candidates, stereotactic radiosurgery can be a suitable alternative for reducing symptoms of diabetes insipidus and neurological deficits [17,18]. In these cases, delivery of high dose be limited by proximity of normal structures like optic nerves, chiasm, brainstem, and cavernous sinus. Combined surgical and postoperative radiation should be considered to treat persistent disease and to reduce risk of tumor progression. Targeted therapies may be suitable adjuvant therapy for susceptible tumors such as HER2-positive breast cancer [19]. The prognosis of pituitary metastasis is generally poor ranging from 6 to 22 months [3].

In our patient, prior to immunohistochemical diagnosis of the surgical specimen, the top differential diagnoses for the pituitary lesion include pituitary adenoma, pituitary carcinoma, metastatic disease, and other non-pituitary origin sellar tumors such as meningioma, epidermoid cyst, germinoma, and chondrosarcoma [20]. Given the patient’s established cancer history, pituitary metastasis should be the highest on the differential until disproven. It is difficult to ascertain whether our patient’s symptoms were due to comorbidities or due to the pituitary metastasis prior to the identification of the lesion on CT imaging. The growth of the pituitary metastasis may have caused the patient’s neurological symptoms due to mass effect or edema, including severe fatigue, headaches, and dizziness. Interestingly, our patient did not develop with diabetes insipidus until after the surgical resection of the tumor. Local edema during post-operative recovery may have contributed to the late-onset of diabetes insipidus as the patient’s symptoms were resolved with prompt desmopressin administration. Recognition of the associated symptoms with pituitary metastases allowed swift management. As described in literature and demonstrated in our patient case, transsphenoidal surgery with adjuvant radiotherapy is a suitable therapeutic option for pituitary metastasis of pulmonary LCNECs.

## Conclusions

Pituitary metastasis is rare, but is especially important to consider in a patient with a known cancer diagnosis. While most patients are asymptomatic at presentation, the presence of diabetes insipidus and/or cranial nerve palsies should raise clinical suspicion of metastatic disease upon radiographic findings of a pituitary lesion. Similar to other pituitary tumors and brain metastases, the preferred management approach of pituitary metastases includes surgery and/or radiosurgery and may incorporate primary-specific systemic or targeted treatments.
